# Clinicopathologic Characterization of Muscle Biopsy Findings in Overlap Myositis: A Single Tertiary Care Center Experience

**DOI:** 10.1002/iid3.70450

**Published:** 2026-04-26

**Authors:** Sidra Fatima, Megha Uppin, Liza Rajasekhar, Reshma Sultana Shaik, Derin Mary Thomas, Phani Kumar Devarasetti

**Affiliations:** ^1^ Department of Pathology Nizam's Institute of Medical Sciences Hyderabad India; ^2^ Department of Rheumatology Nizam's Institute of Medical Sciences Hyderabad India; ^3^ Department of Neurology Nizam's Institute of Medical Sciences Hyderabad India; ^4^ DHR‐ICMR Advanced Molecular Oncology Diagnostic Services Project, Department of Pathology Nizam's Institute of Medical Sciences Hyderabad India

**Keywords:** biomarkers, dermatomyositis, myositis, systemic lupus erythematosus

## Abstract

**Objective:**

This study aims to describe the muscle biopsy features of overlap myositis (OM) and compare them with other inflammatory myopathies. We analyzed clinical, serological findings, and histopathological patterns in muscle biopsies to understand the features of OM.

**Methods:**

Muscle biopsies submitted from patients diagnosed with OM between January 2023 and October 2024 at Nizams Institute of Medical Sciences, Hyderabad were recruited in this study. The clinical and myositis antibody details were noted. The biopsies underwent histological evaluation, including serology, enzyme histochemistry, and immunohistochemistry.

**Results:**

The study cohort included 26 OM patients who underwent muscle biopsy. The female:male ratio is 25:1. The clinical diagnoses of the patients were systemic lupus erythematosus in 12, systemic sclerosis in 5, rheumatoid arthritis in 1, and mixed connective tissue disorder in 2. CPK levels were high in 24 patients. Anti‐Ro52 antibodies were present in 18 patients. Biopsy revealed perivascular inflammation (17), myofiber necrosis (21), and myophagocytosis (7) with infrequent perifascicular atrophy (3). MHC‐I expression was noted in all biopsies.

**Conclusion:**

We report on the muscle biopsy findings in patients with OM. The features align with the ENMC category of “non‐specific myositis” with perivascular inflammation, fiber degeneration and regeneration, and sarcolemmal MHC Class 1 expression being the defining features.

## 1 Introduction

The classification of inflammatory myopathies is evolving. In 2004, the European Neuromuscular Center (ENMC) classified idiopathic inflammatory myopathies (IIMs) into seven categories, such as dermatomyositis (DM), polymyositis (PM), inclusion body myositis (IBM), immune‐mediated necrotizing myopathy (IMNM), ADM (DM sine dermatitis), and non‐specific myositis. **“**Non‐specific myositis” was defined as a subset of IIM that lacks the characteristic histopathologic changes of DM, PM, or IMNM, but still presents with perivascular or perimysial inflammatory cell infiltrates or scattered endomysial CD8+ T cells not clearly surrounding or invading muscle fibers [1]. In 2005, Troyanov et al. [2] proposed the concept of overlap myositis (OM) as a distinct clinical entity. Their study highlighted the use of a clinicoserological approach to the diagnosis of OM [2]. Clinically, OM is defined as “the presence of myositis with at least 1 clinical overlap feature and/or an overlap antibody” [2]. The antibodies considered include those specific for lupus, like anti‐dsDNA and anti‐Sm, those related to scleroderma, like anti‐RNP, anti‐Ro52 antibodies, and the anti‐synthetase antibodies. The clinical overlap features may be one or more features of systemic lupus erythematosus (SLE), systemic sclerosis (SSc), mixed connective tissue disorder (MCTD), rheumatoid arthritis (RA), and Sjögren's syndrome (SS). The prevalence of OM among all inflammatory myositis reported by various studies ranges from 22% to 49% [3]. Recent classification systems emphasize integrated clinicoseropathologic features in inflammatory myopathies [4].

The histopathologic features of many of the subtypes are now well recognized, documented, and incorporated into disease classification criteria. Muscle biopsy is often used to evaluate myositis in these patients, especially when the muscle enzyme levels are normal. In this study, we describe the histopathologic and immunohistochemical features of muscle biopsies from patients with OM and compare them descriptively with those of other inflammatory myopathy subtypes.

## 2 Patients and Methods

### 2.1 Study Design and Population

This is a retrospective single‐center study. In this study, records of OM patients who underwent muscle biopsy during the period from January 2023 to October 2024 at Nizams Institute of Medical Sciences, Hyderabad, Telangana, India, were reviewed. Biopsies reported as DM, IMNM were included as comparators. Medical records of patients fulfilling the criteria for OM who underwent muscle biopsies as part of routine diagnostics for myositis were included. All the biopsies were open biopsies and performed after obtaining written informed consent from the patients. The clinical details were reviewed with medical record files, including clinical presentation and supporting laboratory data, to confirm the diagnosis of the overlap syndrome. Inclusion criteria included recent onset of myopathic symptoms in known patients with a CTD or isolated positivity of a myositis‐associated autoantibody with features of myositis in muscle biopsy. Patients with a characteristic presentation of any subtype of IIM or typical muscle biopsy features of non‐OM myositis or positive myositis‐specific autoantibodies were excluded from the study. Also, the patients with incomplete clinical information or inconclusive autoantibody profiles were excluded from the study. The biopsy slides were reviewed by two pathologists (S.F. and M.U.), with one having more than 20 years of experience in reporting muscle biopsies. The institutional ethics committee allowed waiver of consent for this study (EC/NIMS/3762/2025).

### 2.2 Histological and Immunohistochemical Analysis

The muscle biopsies were open biopsies performed after obtaining written informed consent from the patients. Each muscle biopsy was processed for histochemistry, hematoxylin and eosin (H&E), modified Gomori Trichrome, Oil Red O, enzyme histochemistry (ATPase, SDH, NADH, COX, and COX‐SDH), and immunohistochemistry (IHC) (MHC Class I antigen [1:100, Mouse AntiHLA‐A, Medaysis], MHC Class II antigen [1:100, Mouse AntiHLA‐DR, Medaysis], and Myxovirus Resistant Protein 1 [MX‐1] [1:100, Rabbit mAb, Cell Signaling Technology]) as per the protocol. MHC Class I and Class II staining along the sarcolemma was considered positive. MX‐1 sarcoplasmic staining was considered positive. IHC with CD3, CD20, and CD68 was performed in three biopsies with intense inflammation for cell typing.

Further, the muscle biopsies were systematically evaluated for the following histopathological and immunohistochemical features such as (1) Architecture, (2) Fiber degeneration and regeneration, (3) Perivascular and/or endomysial inflammation‐defined by the presence of inflammatory cells around perimysial blood vessels and around non necrotic muscle fibers, respectively, (4) Presence of necrotic fibers, (5) Perifascicular atrophy (PFA)‐defined by the presence of atrophic fibers in the periphery of muscle fascicles, (6) Immunoexpression of MHC 1 (along the sarcolemma) and MX1 (sarcoplasmic staining). In addition, these features were compared with biopsies of other IIM subtypes, including DM, IMNM, IBM, and AS, during the study period. The biopsy features chosen for analysis are easy to reproduce and form an important part of the objective diagnostic assessment of muscle biopsies. Myositis profile was performed using a 16‐antigen panel (Mi‐2a, Mi‐2b, TIF1g, MDA5, NXP2, SAE1, Ku, PM100, PM75, Jo‐1, SRP, PL‐7, PL‐12, EJ, OJ, and Ro52) by the immunoblot method (EUROIMMUNE Inflammatory myopathy 16 antigen profile). The signal intensity was interpreted as follows: 0–5 (Negative), 6–10 (Borderline), 11–25 (+) (Positive), 26–50 (++) (Positive), and 51–256 (+++) (Strong Positive).

### 2.3 Statistical Analysis

Categorical variables were summarized as frequencies and percentages. Variables with a normal distribution were presented as mean ± standard deviation (SD), while non‐normally distributed variables were expressed as median with interquartile range (IQR; 25th–75th percentile). Accordingly, age at presentation was reported as mean ± SD, whereas creatine phosphokinase (CPK) levels were reported as median (IQR) due to their skewed distribution. The chi‐square (*χ*²) test was employed to explore differences in the distribution of key histological features between patients with OM (*n* = 26) and those with other inflammatory myopathies (*n* = 32). The comparison group included DM (*n* = 10), IMNM (*n* = 10), IBM (*n* = 5), PM (*n* = 3), and anti‐synthetase syndrome (ASS; *n* = 4). Histological features were assessed as binary categorical variables and included fascicular architecture (maintained vs. effaced), myofiber necrosis (present vs. absent), perivascular inflammation (present vs. absent), and perifascicular atrophy (present vs. absent). Statistical significance was defined as a two‐tailed *p*‐value < 0.05. *p‐v*alues between 0.05 and 0.10 were considered borderline significant and interpreted with caution. Effect sizes were interpreted in the context of clinical relevance alongside statistical significance. These analyses were exploratory and descriptive in nature and were not intended to assess diagnostic performance or predictive accuracy of individual histological features. All statistical analyses were performed using GraphPad Prism version 9.5.1 (GraphPad Software, San Diego, California, USA).

## 3 Results

A total of 26 patients (25 female and 1 male) with OM were recruited in this study. The female predominance observed in our cohort is likely related to the underlying disease profile, as most patients had SLE, which is known to occur more commonly in females. The mean age at presentation was 34.9 ± 11.1 years. A well‐defined clinical syndrome was noted in 20 patients: SLE in 12, SSc/scleroderma in 5, RA in 1, and MCTD in 2. In six patients with anti‐Ro52 and inflammatory myositis, no extra‐muscular clinical features were observed.

### 3.1 Clinical Features

All patients were presented with acute to subacute proximal muscle weakness with or without myalgia. CPK levels were elevated in 24 patients with a median of 2183 IU/L (IQR: 334–3811). Baseline demographic, clinical, diagnostic, and biopsy characteristics of the patients are presented in Table 1.

**Table 1:** Clinical and laboratory features of patients diagnosed with overlap myositis (OM) and other inflammatory myopathies.

Sl. No.	Age/Gender	Clinical diagnosis	Clinical features	Duration	Creatine phosphokinase (IU/L)	Serology	Myositis profile	Necrosis/vacuoles	Inflammation	Perifascicular atrophy	Immunohistochemistry
1	42/F	Systemic lupus erythematosus	Muscle weakness	—	2191	ANA speckled 4+, Anti dsDNA −, cytoplasmic 4+ C3‐41, C4‐< 5	—	Scattered necrotic fibers Rimmed vacuoles	Perivascular	—	—
2	27/F	Systemic lupus erythematosus	Pain in thighs, difficulty in getting up, alopecia	4–5 days	244	ANA speckled 4+, cytoplasmic 3+	—	Occasional necrotic fibers	Perivascular	—	MHC1 +
3	52/F	Systemic sclerosis	Weakness of all 4 limbs, difficulty in swallowing	2 years	249	ANA speckled 4+, cytoplasmic 2+	RO52 3+	—	Perivascular	—	MHC1 +
4	30/F	Rheumatoid arthritis	LL proximal muscle weakness, muscle pain	6 months	2800	ANA speckled 4+ cytoplasmic 3+, IgM RF + (97 IU/mL), ANCA‐atypical pattern	RO52 +	—	Endomysial, perivascular	—	MHC1 +, MX1 −, CD3 +, CD20 weak, CD68 strong
5	21/F	Systemic lupus erythematosus	Truncal weakness, thigh, and arm muscle tenderness	—	40,000	ANA 4+, Anti dsDNA − C3‐38 C4‐4	KU 3+	Scattered necrotic fibers	Perivascular	—	MHC1 +, MX1 −
6	32/F	Mixed connective tissue disorder	Muscle weakness and pain	—	3811	—	RO52 3+	—	Perivascular	—	MHC1 +, MX1 −, CD3 +, CD20 positive in aggregated CD68 strong
7	20/F	Systemic lupus erythematosus	Truncal B/L UL and LL weakness, skin rashes	3 weeks	943	ANA nucleolar 4+, Anti dsDNA + (4.3), ACL IgM 59, IgG 42, Anti Sm +, C3‐27, C4‐3	RO52 2+, KU 2+, MDA5 2+, TiFa +, Mi2a +	Scattered necrotic fibers Rimmed vacuoles		Present	MHC1 +, MX1 −
8	45/F	Systemic sclerosis	Persistent limb and axial muscle weakness	—	85	ANA speckled 4+, Anti dsDNA −	RO52 +	Many necrotic fibers Rimmed vacuoles		—	MHC1 +
9	24/F	Systemic lupus erythematosus	Weakness of arms and legs, fever	1 month	1400	ANA 4+, Anti dsDNA +(1.2), ACL IgM 28, ANCA‐atypical pattern, Anti Sm weak + (38U), C3‐45, C4‐6	RO52 3+, Mi2a+, Mi2b+, MDA5 +, KU +, SRP +, PL7 +, PL12 +, EJ +, OJ+	Many necrotic fibers	Perivascular	—	MHC1 +, MX1 −
10	26/F	Systemic lupus erythematosus	Proximal muscle weakness, Gottron's sign	—	183	ANA speckled 4+, Anti dsDNA borderline + (0.92), cytoplasmic 3+ C3‐63, C4‐7	RO52 3+, PL12 +, PL7 +, Mi2b +	Scattered necrotic fibers	Perivascular	—	
11	37/F	Systemic lupus erythematosus/Dermatomyositis	Proximal muscle weakness, alopecia, hyperpigmented scaly lesions	7 months	3134	ANA speckled 4+, Anti dsDNA −. Cytoplasmic 3+ C3‐105, C4‐16	RO52+, MDA5 +	Scattered necrotic fibers	Perivascular	—	MHC1 +, MX1 −
12	56/F	Scleroderma/Polymyositis/Dermatomyositis	Neck pain, difficulty in squatting, dysphagia	—	2918	—	RO52 3+, KU 3+, Mi2a +	Occasional necrotic fibers	—	—	MHC1 +, MX1 −
13	40/F	Inflammatory myopathy	Proximal muscle weakness, truncal and neck weakness	3 months	> 5000	ANA speckled 4+, Anti dsDNA −, ANCA‐atypical pattern, IgM RF + (391 IU/mL), C3‐152, C4‐56	RO52 3+	Scattered necrotic fibers	Endomysial, Perivascular	—	MHC‐1 + MX‐1 − CD3 + CD20 in aggregates CD68 strong
14	34/F	Systemic sclerosis	Pain in the B/L thighs and calves, difficulty in getting up	2 months	8557	ANA speckled 4+	NXP2 +	Scattered necrotic fibers	Endomysial	—	MHC1 +, MX1 −
15	33/F	Systemic lupus erythematosus	Proximal UL and LL weakness	1 month	23	ANA speckled 4+, cytoplasmic 2+, Anti dsDNA −, C3‐80, C4‐5	RO52 +	Scattered necrotic fibers	Perivascular	Present	MHC1 +, MX1 +
16	23/F	Systemic lupus erythematosus	Proximal muscle weakness	—	3779	ANA speckled 4+, AntidsDNA + (350 IU/mL) cytoplasmic 3+, C3‐83, C4‐< 5	NXP2+, KU+	—	—	—	MHC1 +
17	27/F	Systemic lupus erythematosus	Malar rash, alopecia, facial pigmentation	—	1635	ANA speckled 4+, cytoplasmic 3+, Anti dsDNA −, C3‐22, C4‐13	NXP2+, SAE1+	Scattered necrotic fibers	—	—	MHC1 +, MX1 −
18	37/F	Mitochondrial myopathy/Overlap myositis	Arthritis, right eye ptosis, neck UL and LL weakness	6 months	2175	ANA speckled 4+, cytoplasmic 3+, Anti dsDNA −, C3‐92, C4‐3	RO52 +	Few necrotic fibers	Endomysial, Perivascular	—	MHC1 +, MX1 −
19	22/F	Systemic lupus erythematosus	Proximal muscle weakness, vasculitic rash, alopecia	—	334	ANA nucleolar 4+, AntidsDNA + (409 IU/mL), C3‐95, C4‐17	NXP2+, RO52+	Dominant necrosis	Perivascular	Present	MHC1 +, MX1 +
20	47/F	Immune‐mediated necrotizing myopathy/Polymyositis	Proximal muscle weakness, bulbar, and axial weakness	1 month	194	ANA speckled 3+, AntidsDNA −, C3‐145, C4‐29	RO52 3+, SRP +, PL7 +	Dominant necrosis	—	—	MHC1 +, MX1 −
21	48/F	Unspecified myositis	B/L Carpal tunnel syndrome	6 months	5456	—	RO52 3+	—	Endomysial, Perivascular	—	MHC1 +, MX1 −
22	55/F	Systemic sclerosis	Proximal muscle weakness LL > UL, salt and pepper pigmentation, ILD	20 days	3175	ANA cytoplasmic dense fine speckled 4+, Anti dsDNA −, C3‐141, C4‐16	RO52 3+	Occasional necrotic fibers	—	—	MHC1 +, MX1 −
23	47/F	Dermatomyositis, CAM (k/c/o high‐grade serous ovarian carcinoma)	Subacute quadriparesis with proximal predominance	1 month	8864	ANA mitochondrion like pattern 4+	RO52 3+	Occasional necrotic fibers Rimmed vacuoles	Perivascular	—	MHC1 +, MX1 −
24	35/F	Unspecified myositis	Muscle pain and weakness	3 months	2000	Anti Sm/RNP 3+	SRP 2+, CN‐1A +	Scattered necrotic fibers	—	—	—
25	31/F	Systemic lupus erythematosus	Difficulty in getting up from a sitting position, Proximal muscle weakness	—	7227	ANA speckled 4+, cytoplasmic 3+, AntidsDNA −, ANCA‐atypical pattern, IgM RF+ (61 IU/mL), C3‐77, C4‐8	—	Many necrotic fibers	Endomysial, perivascular	—	MHC1 +, MX1 −
26	17/M	Mixed connective tissue disorder	Difficulty in swallowing, pain in thigh muscles, difficulty in squatting	4 months	> 1000	—	RO52 +, RNP SM+3	Scattered necrotic fibers	Perivascular	—	MHC1 +, MX1 −

*Note:* Continuous variables are expressed as mean ± standard deviation or median (interquartile range), as appropriate. Categorical variables are expressed as frequencies and percentages.

Abbreviations: CPK, creatine phosphokinase; IQR, interquartile range; OM, overlap myositis.

### 3.2 Myositis Autoantibodies

Myositis autoantibodies were assessed in 23 patients. Anti‐Ro52 was the most frequently detected autoantibody, present in 18 patients, followed by anti‐Ku, which was detected in 5 patients.

### 3.3 Muscle Biopsy Features

#### 3.3.1 Histopathology

**Figure 1:**
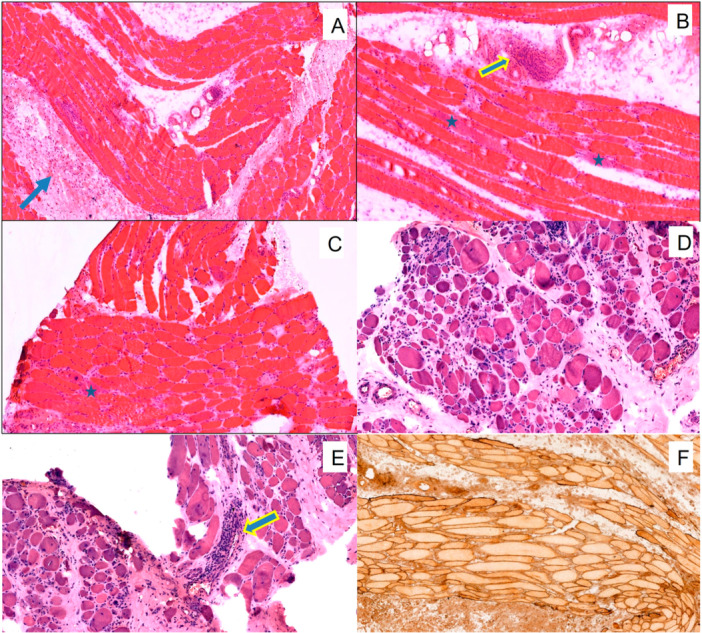
Representative histopathological and immunohistochemical features on muscle biopsy. Representative muscle biopsy images demonstrating: (A) Hematoxylin and eosin (H&E)–stained section showing preserved fascicular architecture with marked interstitial edema (blue arrow); (B) perivascular lymphocytic inflammation (yellow arrow); (C) prominent myofiber regeneration (asterisks); (D and E) fibrosis identified in a single biopsy specimen in association with active perivascular inflammation (arrow) and regenerating fibers; and (F) immunohistochemistry (IHC; ×40) showing diffuse sarcolemmal overexpression of major histocompatibility complex (MHC) Class I in muscle fibers.

The muscle architecture was maintained in all patients' biopsies except one, where the muscle was replaced by fibrosis and adipose tissue. This patient had a myopathy of more than 3 years duration. All the muscle biopsies presented significant fiber degeneration and regeneration. Inflammation (perivascular and/or endomysial, as defined under histological analysis) was identified in 18 biopsies. It was predominantly perivascular in 17 biopsies and endomysial (around non‐necrotic fibers) in 8. Marked inflammation with lymphoid aggregates in the perimysial region, extending into the endomysium, was observed in three biopsies. There was no vascular destruction or fibrinoid necrosis in any of the biopsies. The photomicrographs illustrating various biopsy features in these patients are shown in Figure 1.

**Figure 2:**
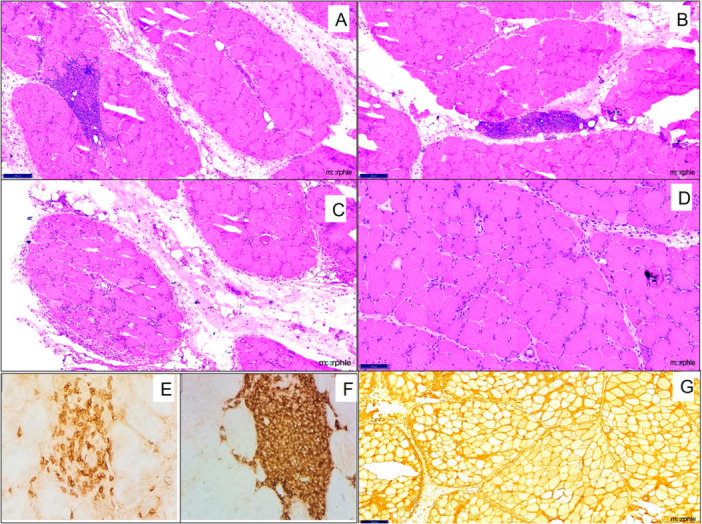
Muscle biopsy findings in a 30‐year‐old female with systemic sclerosis. Representative muscle biopsy images showing (A and B) dense perifascicular and endomysial inflammatory infiltrates; (C) interstitial muscle edema; (D) scattered necrotic myofibers; (E) immunohistochemistry (IHC; ×40) for CD3 demonstrating membranous positivity in T lymphocytes; and (F) IHC (×40) for CD20 highlighting lymphoid aggregates; (G) Diffuse sarcolemmal overexpression of MHC Class I is also noted on IHC (×40).

**Figure 3:**
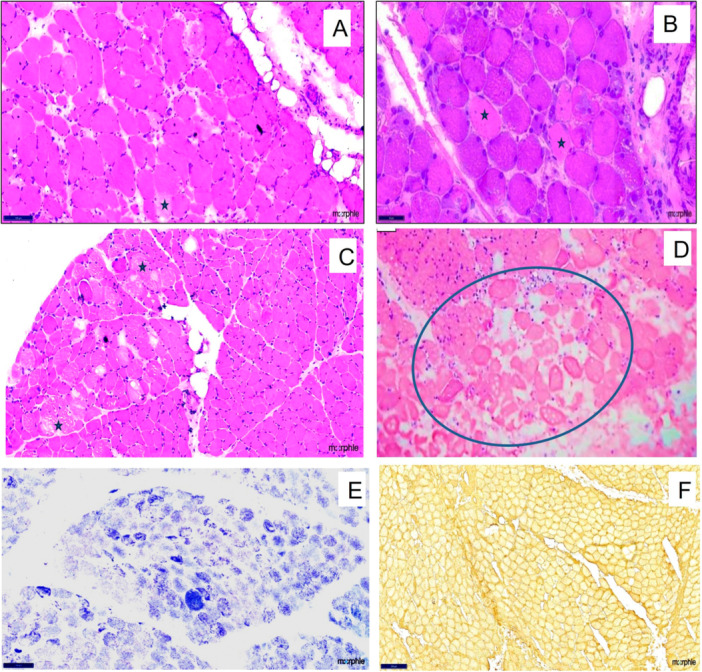
Muscle biopsy findings in a 20‐year‐old female with systemic lupus erythematosus. Representative muscle biopsy sections showing (A and B) H&E (×100) – stained sections with scattered necrotic myofibers (asterisks); (C) H&E (×100) demonstrating necrotic fibers with associated perifascicular atrophy; (D) H&E showing groups of necrotic fibers forming microinfarct‐like areas; (E) nicotinamide adenine dinucleotide (NADH) stain (×100) highlighting moth‐eaten fibers; and (F) immunohistochemistry (IHC; ×100) showing diffuse sarcolemmal expression of MHC Class I.

**Figure 4:**
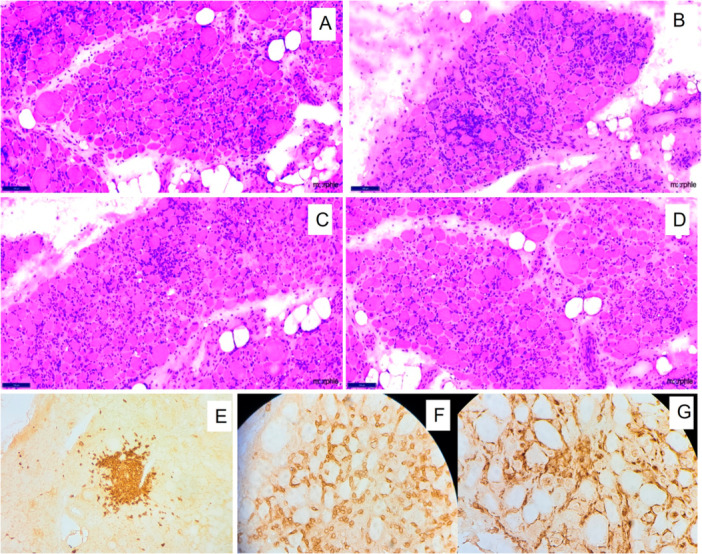
Muscle biopsy findings in a 32‐year‐old female with mixed connective tissue disease. Representative muscle biopsy images demonstrating (A–D) H&E‐stained sections (×100) showing dense endomysial inflammation with formation of small lymphoid aggregates; (E) immunohistochemistry (IHC; ×40) for CD20 highlighting lymphoid aggregates; (F) IHC (×40) for CD3 showing membranous positivity in T lymphocytes; and (G) IHC (×40) for CD68 highlighting numerous scattered histiocytes in the endomysium.

Myofiber necrosis was predominant in 21 muscle biopsies and seen in 11 of 12 biopsies in patients with SLE. The necrosis was scattered in 7 out of 12 biopsies, and large areas of grouped infarct‐like necrosis were present in 5. Infiltration of necrotic fibers by phagocytes (myophagocytosis) was observed in seven biopsies. Rimmed vacuoles were seen in four, and perifascicular atrophy was seen in only three cases. Microphotographs highlighting the biopsy findings in various index patients and their underlying overlap conditions are shown in Figures 2, 3, 4.

Enzyme histochemical stains revealed abnormal mitochondrial blue ragged fibers in one biopsy. The checkerboard pattern was maintained in all, and Masson trichrome revealed fibrosis in one. Comparison of key histopathological features between OM and other inflammatory myopathy subtypes (Table 2).

**Table 2:** Breakdown of inflammatory myopathy subtypes included in the non‐overlap myositis comparison group.

**Biopsy features**	Polymyositis	Dermatomyositis	Inclusion body myositis	Immune‐mediated necrotizing myopathy	Anti‐synthetase syndrome
*n*	**3**	**10**	5	10	**4**
Architecture	Maintained	Maintained	Maintained to efface	Maintained	Maintained
Necrosis	Absent	Scattered (More in NXP2 DM)	Absent	Majority	Scattered (*n* = 3), Perifascicular (*n* = 1)
Inflammation	Endomysial around non‐necrotic fibers	Perivascular	Endomysial with rimmed vacuoles	Minimal to absent	Endomysial and perivascular
Perifascicular atrophy	Absent	Present (*n* = 7), Absent (*n* = 3)	Absent	Absent	Present (*n* = 2)
MHC Class I along sarcolemma	Positive	Positive	Positive	Positive in non‐necrotic fibers	Positive
Myxovirus‐Resistant Protein‐1	Negative	Positive (*n* = 8), Negative (*n* = 2)	Negative	Negative	Negative
MHC Class II	Negative	Positive (*n* = 2), Negative (*n* = 7)	Negative	Negative	Positive (*n* = 3, perifascicular), Negative (*n* = 1)

Abbreviations: ASS, anti‐synthetase syndrome; DM, dermatomyositis; IBM, inclusion body myositis; IMNM, immune‐mediated necrotizing myopathy; PM, polymyositis.

#### 3.3.2 IHC

Diffuse expression of MHC‐I along the sarcolemma was present in all cases. MX‐1 expression was present in 2 of 19 biopsies in which it was done. Both patients had SLE. There was no PFA or DM associated myositis specific antibody in these two patients. The staining was minimal rather than the strong perifascicular expression, which is commonly seen in DM. 3 biopsies with marked inflammation were subjected to B and T cell typing. The endomysial cells were all T cells (bright CD3 positivity), whereas the perimysial ones were B cells, positive for CD20. In addition, these biopsies showed extensive histiocytic infiltrate marked by positive CD68.

### 3.4 Clinicopathologic Correlation

Key histological features—including fascicular architecture, myofiber necrosis, perivascular inflammation, and perifascicular atrophy—were compared between patients with OM (*n* = 26) and those with other inflammatory myopathies (*n* = 32). Perivascular inflammation was significantly more frequent in OM compared with other inflammatory myopathies (*χ*² = 5.524, *p* = 0.0188). The presence of myofiber necrosis did not show a statistically significant association with OM, although a borderline association was observed (*χ*² = 3.068, *p* = 0.0799). Further analysis of necrosis patterns demonstrated a significant association between OM and scattered necrotic fibers (*χ*² = 6.776, *p* = 0.0092). In contrast, the presence of groups of necrotic fibers did not show a significant association with OM (*χ*² = 0.002, *p* = 0.9605). No significant differences were observed between the two groups with respect to perifascicular atrophy (*χ*² = 2.405, *p* = 0.1209) or effacement of muscle fascicular architecture (*χ*² = 0.169, *p* = 0.6810). The findings are summarized in Table 3.

**Table 3:** Comparison of selected histopathological features on muscle biopsy between patients with overlap myositis and other inflammatory myopathies.

Feature	*χ*² value	*p* value	Statistical significance (*p* < 0.05)
Architecture	0.169	0.6810	Not significant
Necrosis	3.915	0.0479	Significant (borderline)
Perivascular inflammation	5.524	0.0188	Significant
Perifascicular atrophy	2.405	0.1209	Not significant

*Note:* Histological variables were assessed as binary categorical parameters. Statistical comparisons were performed using the chi‐square (*χ*²) test. Statistical test: *χ*² test for categorical variables.

## 4 Discussion

In our study cohort, the majority of the patients had an SLE myositis overlap. The SLE myositis and muscle biopsy have been studied in previous reports emphasizing muscle involvement, often presenting as diffuse pain or muscular weakness. Liang et al. [5] investigated a cohort of 1701 SLE patients, observed myositis in 44 patients, and reported muscle necrosis and inflammation as the most common features in SLE myositis muscle biopsies. Tiniakou et al. studied 16 muscle biopsies performed in 179 SLE patients. These biopsies were reported as necrotizing myopathies (50%) and consistent with DM due to the presence of PFA (38%) [6].

The observation of muscle fiber necrosis in SLE myositis closely aligns with previous reports, particularly with respect to clinical presentation and biopsy features [6, 7]. To understand the association of inflammatory myositis (and SLE myositis, itself) with the presence of necrosis on muscle biopsies, explanations have been put forward by a few studies. Muscle cell death in myositis does not result from apoptosis, unlike that seen in muscular dystrophies. Nagaraju et al. explained the possible mechanism of injury and cell death in patients of inflammatory myopathies by observing the presence of FLICE (Fas‐associated death domain‐like IL‐1‐converting enzyme)‐inhibitory protein (FLIP). FLIP acts as a mechanism of resistance to apoptosis in muscle cells despite the presence of Fas and FasL in inflamed tissue. This could explain how the cells evade Fas‐mediated apoptosis and choose to die by necrosis instead [8].

In another study by Li and Dalakas, the presence of human IAP‐like protein (hILP) on the muscle membrane in patients with inflammatory myopathies was thought to contribute to the evasion of apoptosis. hILP is a cell death suppressor that inhibits executioner caspases. This finding suggested a possible mechanism for the lack of apoptosis in inflamed muscle fibers [9].

Both these studies were conducted on muscle biopsies using methods of laser capture microscopy, immunocytochemistry, immunoblotting, and subcellular fractionation.

It is recently understood that certain Regulated Cell Death (RCD) processes in muscle tissue could be a possible therapeutic target in patients with myositis [10]. Kamiya et al. discussed the role of necroptosis in the pathophysiology of IIM, emphasizing its potential as a target for treatment strategies in these patients. Inhibition of certain damage‐associated molecular patterns from injured muscle fibers was found to ameliorate muscle weakness in patients with IIM, proposing therapeutic advances apart from regular immunosuppression, which can have infection‐related side effects [7, 11]. They have also mentioned the details of clinical trials based on necroptosis inhibitors in a tabular format. These studies have focused on IIMs; however, similar mechanisms could operate in OM. It remains an important area for future research to establish whether any of these mechanisms are upregulated in SLE‐associated myositis, contribute to muscle necrosis, and could be leveraged for targeted therapeutic intervention.

Muscle necrosis is a dominant feature of necrotizing myopathies. These include immune (IMNM) and nonimmune mechanisms as seen in rhabdomyolysis, critical illness myopathy, toxic myopathy, and muscular dystrophies. Merlonghi et al. [12] in their meta‐analysis on myopathology of necrotizing myopathies have compared the muscle biopsy findings between inflammatory and non‐inflammatory (nIM) myopathies. Necrosis was found to be more frequent and statistically significant in IMNM compared to DM, ASS, and nIMs. However, OM was not a part of this analysis. We identified scattered necrosis to be more frequent in OM as compared to the non‐OM group in our study. However, this requires validation in a larger cohort including OM and IMNM. To avoid misdiagnosis of IMNM versus nIM, it is important to correlate the muscle biopsy findings with clinical details, serology, imaging, and IHC for an appropriate diagnosis.

Lepreux et al. [13] also reported on the role of muscle biopsy in IIMs overlapping with systemic diseases. They observed that patients with myositis‐associated antibodies (MAAs), namely anti‐Ro, anti‐La, anti‐KU, anti‐PM/Scl, and anti‐U1snRNP, had a non‐specific myositis pattern on muscle biopsy and often had SSc, SLE, RA, SS, and MCTD. The muscle biopsy findings in these patients uniformly included myofiber degeneration/regeneration and damage with a degree of endomysial/perimysial/perivascular inflammatory mononuclear cell infiltrates. Since inflammation, fiber degeneration, regeneration, and necrosis are common histopathologic findings in IIM subtypes, careful evaluation of these individual characteristics in any biopsy is essential for an accurate histopathologic diagnosis. The systematic analysis in our study has emphasized the presence of perivascular inflammation and necrosis as being more frequent in OM.

Siegert et al. [14] studied muscle biopsies in 18 patients with SSc by immunohistochemical analysis and electron microscopy. It was found that 12 of these 18 patients showed minimal features of myositis on biopsy but had clear capillary alterations, termed minimal myositis with capillary pathology. The biopsies showed sparse endomysial T‐lymphocytic infiltrates and the absence of any muscle fiber invasion, with necrotic fibers being exceptional. In contrary to this observation, our biopsies demonstarted increased inflammation. However, the capillary pathology was not evaluated.

While mentioning SSc to be the commonest overlap syndrome in myositis. Lepreux et al. [13] reported heterogeneous biopsy features ranging from inflammation, fiber degeneration/regeneration to overt necrosis. Similar observations have been reported from a John Hopkins study including 42 patients of scleroderma [15]. Fiber necrosis was a common feature in the biopsies of scleroderma, with necrotizing myopathy pattern seen in 28 of the 42 patients (66%). Statin exposure was recorded in only two patients.

These findings align with the features noted in SSc/scleroderma patients in our cohort, with all but one biopsy showing the presence of fiber necrosis and perivascular inflammation. PFA, a defining feature of DM, has been reported in patients with OM. Troyanov et al. [16] noted that out of the 10 biopsies that showed PFA, 6 were in the overlap group and did not have a DM rash at diagnosis. They defined the entity of OM with DM features. These cases have evidence of PFA on muscle biopsy but no DM specific rash and have the presence of overlap autoantibodies such as anti‐synthetases (Jo1, PL7), anti‐PM/Scl, anti‐U1RNP, and anti‐Ro52. Other experts now believe ASS to be a distinct subtype of IIM [17]. In our study, PFA was observed in 3 biopsies. These cases were positive for Ro52 antibodies and had an SLE‐myositis overlap, thereby supporting a diagnosis of OM over DM.

In DM, along with scattered necrotic fibers and PFA, inflammation mainly occurs around blood vessels and interfascicular septae, with B cells, CD4+ T cells, dendritic cells, and macrophages, while CD8+ T cells and NK cells are rare [18]. In our study, we observed inflammation to be predominantly perivascular (*n* = 17) but also with foci of endomysial inflammation in a significant number of cases (*n* = 8) with necrosis ranging from scattered to large groups of necrotic fibers. Cell typing was done for three biopsies with extensive inflammation, highlighting CD3‐positive T cells, CD20‐positive B cells, and CD68‐positive macrophages, showing a cell population like DM.

Muscle biopsies in patients with overlap syndrome are performed in the acute/subacute phase of muscle weakness, and hence the studies concentrate on histologic features of activity, such as necrosis, inflammation, and regeneration. Fibrosis is a rare finding and has been peculiarly shown to be associated with PM‐Scl antibodies [15]. Fibrosis was observed only in a single patient of scleroderma in our series; however, it was associated with active inflammation, regeneration, and necrosis.

Type 1 interferon–inducible genes, including MX‐1, have been linked to DM, with perifascicular or diffuse MX‐ 1 overexpression observed in muscle biopsies. It has been established that sarcoplasmic MX‐1 is a more sensitive marker for diagnosing DM than traditional markers [19]. In our study, only 2 patients showed positivity for MX‐1. Both cases had SLE. Xing et al. [20] have reported a high prevalence of MX‐1 expression in lupus myositis and mention this positivity as an important marker that could be used to differentiate lupus myositis from IMNM and ASS.

In IMNM, the hallmark feature is random muscle fiber necrosis with regeneration, along with minimal or no mononuclear cell infiltrates. In addition, the sarcolemma of muscle fibers does not express MHC Class I antigen [18]. In our study, although necrotic fibers have been observed, the majority of the patients showed significant inflammatory cell infiltrates, and all our patients expressed diffuse positivity for MHC I.

MAAs were found in patients with myositis and other autoimmune diseases. Unlike myositis‐specific antibodies (MSAs), which are unique to IIMs, MAAs can occur in patients with myositis as well as in those with other connective tissue diseases. Selva‐O'Callaghan et al. studied the prevalence of MSA and MAA in patients with IIM and reported that among the MAA, the most detected were anti‐Ro60/52, anti‐PM/Scl, anti‐Ku, and anti‐U1RNP. These patients were more commonly identified as having overlap syndrome [21]. Similarly, Ghirardello et al. studied autoantibodies like anti‐Ro/SSA, anti‐PM/Scl, anti‐Ku, and anti‐U1RNP. They reported that these MAA are often found when PM or DM occurs with another connective tissue disease, indicating overlap syndrome [22].

Myositis on muscle biopsy is only infrequently reported in patients of Sjogren's and RA, though many can have muscle pain and mild weakness [13].

## 5 Limitations of the Study

The retrospective and observational nature of the study, with a limited sample size, are certain limitations of the present study.

## 6 Conclusion

In this study of muscle biopsies in patients with OM, we suggest that ENMC‐defined non‐specific myositis is the most common pathology. Prominent myofiber necrosis, particularly in SLE‐associated myositis, and frequent perivascular inflammation were the dominant features in our cohort. These findings expand the understanding of muscle pathology in OM and reinforce the importance of integrated clinicopathologic and serologic correlation in routine practice. Future prospective studies with larger cohorts may help to assess the diagnostic significance in seronegative patients.

## Author Contributions

All authors have contributed towards the collection of patient samples and data, the design and experiments of the study. D.M.T. contributed to the preparation of the final manuscript.

## Funding

The authors received no specific funding for this study.

## Ethics Statement

This is a retrospective descriptive study. The muscle biopsies and other investigations mentioned in the study are a part of the routine diagnostic workup. Written informed consent was obtained from every patient before the procedure. No additional investigations have been done. The study reflects an analysis of the retrospective data to put forth our experience of diagnosis of Overlap Myositis.

## Conflicts of Interest

The authors declare no conflicts of interest.

## Supporting information

Supporting File

## Data Availability

The authors have nothing to report.
